# TALEN based HPV-E7 editing triggers necrotic cell death in cervical cancer cells

**DOI:** 10.1038/s41598-017-05696-0

**Published:** 2017-07-14

**Authors:** Sumitra Shankar, Deepti Prasad, Rahul Sanawar, Ani V. Das, M. Radhakrishna Pillai

**Affiliations:** Cancer Research Program, Rajiv Gandhi Centre for Biotechnology, Thycaud. P. O., Thiruvananthapuram-14, Kerala, India

## Abstract

Human Papillomavirus E7 and E6 oncoproteins have been considered as suitable candidate anti-viral targets since they cause malignant conversion in cervical cancers. Transcription Activator-Like Effector Nucleases (TALENs) are recent editing tools to knockout genes by inducing double stranded breaks at specific sites in the genome. In here, we have designed specific TALENs to target E7 and analyzed their efficiency in inducing cell death in cervical cancer cells. We found that designed TALENs could yield about 10–12% editing activity as observed from T7E1 and nuclease resistance assays. Down-regulation of E7 and E6 was further evident at the transcript as well as proteins levels indicating that the selected TALENs were effective. TALEN-mediated E7 editing led to cell death as ascertained by cell cycle and Annexin V assays. Annexin profiling suggested that cell death could be due to necrosis as observed by upregulation of necrotic markers such as LDH A, Rip-1, and Cyclophilin A. Necrosis appears to be a better therapeutic response as it could further activate pro-inflammatory cytokines to attract immune cells to eliminate HPV-integrated cells and therefore TALEN editing strategy has the potential to be a promising tool as an adjuvant therapy in cervical cancer along with surgery.

## Introduction

Human Papilloma Virus (HPV) is the most important risk factor for cervical cancer. Though most of the HPV infections clear up spontaneously, in some women HPV infection may progress to invasive cervical cancer. It is observed that HPV infection is common in sexually active women in their early 20s and development of cervical cancer is prominent in older women suggesting that persistent HPV infection gradually progresses to cancer^1^. Of the types that are associated with cervical cancer, HPV 16 and 18-induced malignancies are the most predominant high-risk types worldwide. Moreover, HPV subtype 16 is the most predominant oncogenic type in India^2^.

As E6 and E7 are the primary HPV 16 oncogenes involved in transformation, they have been targeted using a range of approaches including siRNA, ribozymes and peptides^3, 4^. These, however, only suppress their actions. In this context, genome editing using synthetic nucleases offers an advantage over other approaches such that a specific area of the DNA could be modified with a single dose of administration of these molecules, thereby editing the gene of interest at once and making it non-functional. Initial studies have shown that when the DNA binding domain of the bovine papillomavirus type 1 (BPV1) E2 protein was fused to the catalytic domain of the FokI restriction endonuclease, it generated a BPV1 E2-FokI chimeric nuclease (BEF) which could introduce DNA double-strand breaks on E2 binding site *in vitro*, and also cleaved its target DNA in HeLa cells^5^. Upon introduction into the cell, programmable nucleases trigger targeted chromosomal breaks *in vivo* and stimulate either homologous recombination or non-homologous end joining (NHEJ). In this error-prone repair pathway, the cut DNA ends are quickly joined back together, mostly with minor deletions or additions at the break site, thereby disrupting the coding sequence of the gene. Thus, it became possible to precisely edit any gene of interest^6, 7^. Zinc finger nucleases (ZFNs) and transcription activator-like effector nucleases (TALENs) are two such molecules.

TALENs are fusion modular proteins composed of an N-terminal translocation domain, central repeats that together mediate sequence-specific DNA binding and a C-terminal segment that contains nuclear localization signals (NLS) joined with FokI endonuclease^8^. The central TALE DNA binding domain contains conserved 33–35 residues long repeats arranged in tandem arrays^9–11^. TALENs have attracted the researchers since their designing is simpler and easier than that of ZFNs. TALEN based studies have shown to eliminate infection caused by other viral systems such as HCV, HBV and HIV. It has been previously reported that DGAT1, a host factor responsible for assembly of HCV, was silenced by TALEN delivery and impairment of HCV entry was down-regulated by Claudin expression^12^. Other researchers have analyzed anti-viral activity of TALEN targeting HBV genome and detected TALEN-induced mutations in Covalent Circular Closed DNA of HBV^8, 13^. Similarly, TALENs were also used to target the HIV-1 LTR *in vitro* ^14^. Studies have also shown that they have been used to correct genetic diseases such as sickle cell disease^15^ and *xeroderma pigmentosum* ^16^. In a similar fashion, HPV associated genes have also been targeted with TALENs.

In the present study, we investigated the mechanism of cell death involved after editing E7 gene with TALENs. TALENs targeting E7 was assessed by endonuclease assays and immunofluorescence experiments. E7 editing was further corroborated by RT-PCR, western blot and FACS analyses. Finally, cell cycle and Annexin V assays indicated necrosis which was confirmed by upregulation of necrotic markers such as LDH-A, RipI and Cyclophilin A.

## Results

### TALEN design and construction against E7 gene of HPV 16 genome

**Figure 1:**
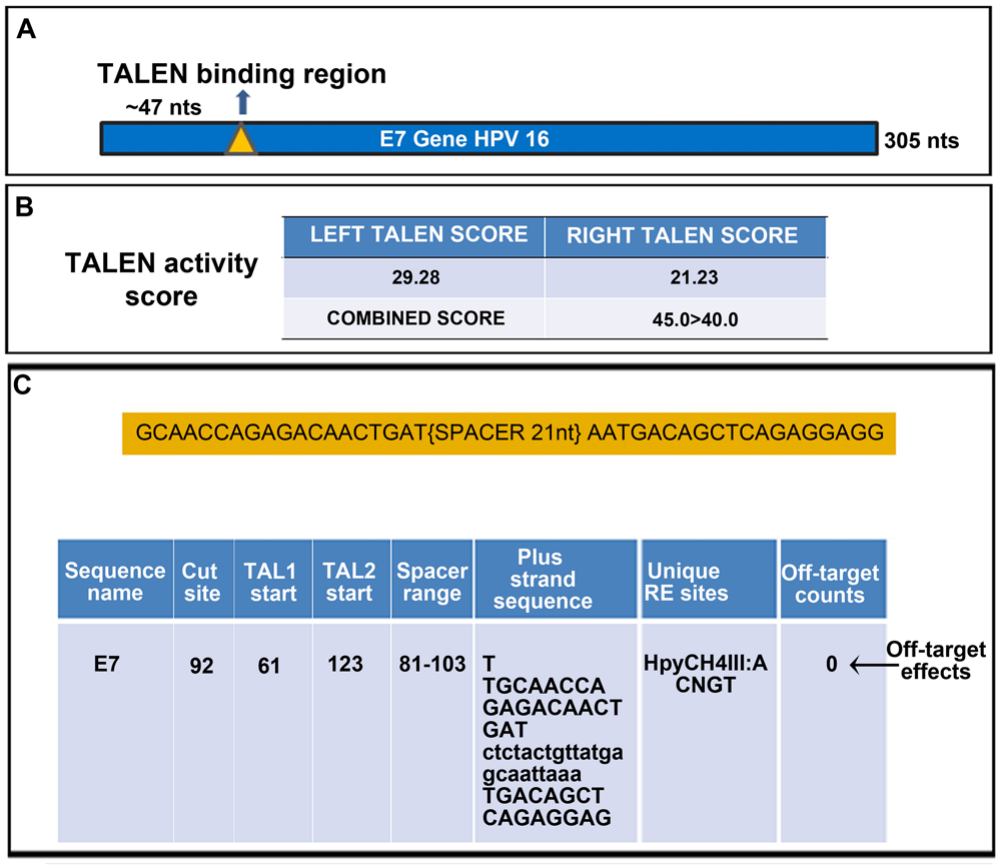
Designing of TALENs targeting the HPV 16 E7 gene. (**A**) Schematic of TALEN binding site on E7 gene. (**B**) TALEN predicted activity using SAPTA software. (**C**) Part of E7 sequence targeted by TALEN.

For gene disruption, the first criterion is that targeting coding exons towards beginning of the gene may create mutations leading to complete disruption of the gene and be less likely to generate truncated protein artifacts with residual biological activity. The second criterion is to screen for unique areas in the genome in order to minimize off-target effects^17^. Taken these criteria into consideration, TALEN pairs were screened using SAPTA software and the region selected was such way that it targeted at position 44 which was in the start region of Exon I of E7 (Fig. 1A and B). This TALEN pair had 19 binding sites on either side with a spacer region which was of about 21 nucleotides (Fig. 1C). Our analysis suggested that the TALEN pairs we selected were appropriate for using in the gene editing experiments. Thus, the TALEN pair was synthesized and sequenced which matched the TALEN designed for this particular site (Supplementary Fig. 1).

### Selected TALEN pairs were effective in E7 editing in SiHa cells

**Figure 2:**
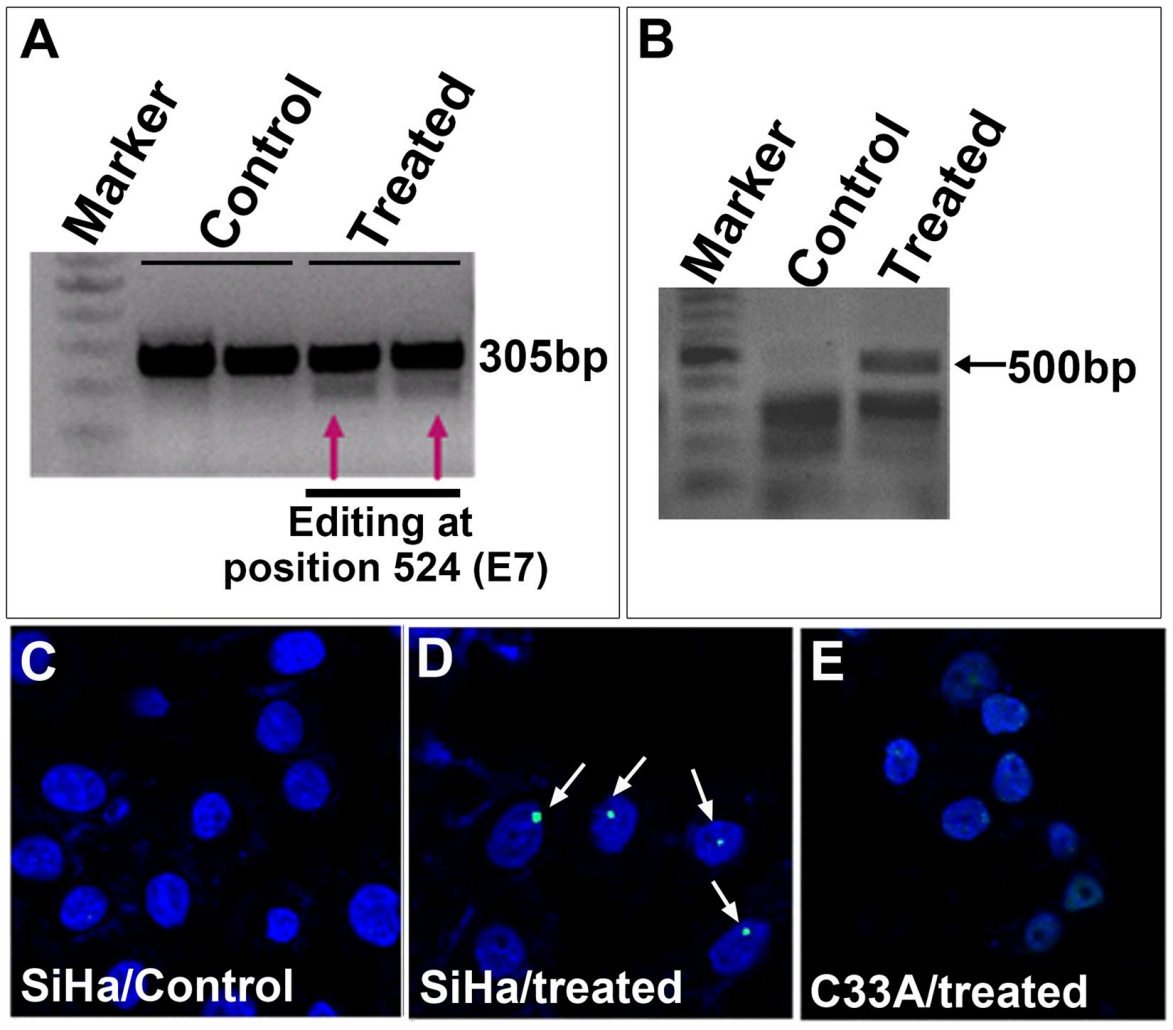
Selected TALEN pair could effectively target E7 in SiHa cells *in vitro*. In order to check the designed TALENs were able to target HPV-E7, SiHa cells were transfected with TALENs and endogenous gene disruption of E7 gene was assessed by (**A**) T7E1 assay indicating a cut product at 258 bp size run on a 2% agarose gel, and (**B**) Nuclease resistance assay showing a product of 500 bp obtained upon digestion with HpyChIII on E6-E7 gene of size 775 nucleotides. Immunocytochemical analysis showed the presence of 53bp1 in TALEN-treated SiHa cells (**D**) which was absent in SiHa control cells (**C**) and HPV^−ve^ C33A cells (**E**). Magnification 200X.

In order to check whether the designed TALEN pairs were effective in gene editing of E7 gene of HPV, we transfected SiHa cells with the TALEN pair targeting E7 from position 44 to 103 nt. SiHa cell line was chosen because it has only a single HPV 16 genome in it. Transfection efficiency in SiHa cell line was monitored using vector containing GFP. We obtained around 70% efficiency for transfection in SiHa cells. After seventy-two hours of treatment, genomic DNA was extracted and editing was analyzed by various methods. First, we checked the editing by using T7E1 assay. E7 gene is 305 bp. The genomic DNA was subjected to digestion with T7E1 enzyme which could cut at position 47 and generate a band of size 258 bp as seen in Fig. 2A. Next, we performed complementary nuclease resistance enzyme assay using HpyCh4 III which cut at the same position as that of TALENs. If editing has happened this enzyme would not be able to cut at the same position and therefore will not be able to generate excised bands. Our results showed that in treated cells editing had happened and therefore were unable to generate bands of expected size upon digestion with HpyCh4 III, whereas bands were present in controls (Fig. 2B). Using Image J software, the band intensities were measured as peaks and the area under them was then calculated. We observed approximately 10% editing of E7 gene in SiHa cells for T7E1 and 12% NR assays (Supplementary Fig. 2). E7 editing was further corroborated by doing immunocytochemical analysis for 53BP1 which marks for double strand breaks in the DNA^18^. Thirty six hours after TALEN treatment, SiHa cells were subjected to immunostaining for 53BPI antibody. Only treated cells showed a single green spot representing the presence of 53bp1 thus indicating the effect of TALEN in those cells (Fig. 2D), while the control cells did not show such pattern (Fig. 2C). In order to check the specificity, we selected another cervical cancer cell line, C33A, which did not have HPV integrated in the genome. C33A cells were also treated with TALENs and immunocytochemical analysis with 53BP1 antibody was carried out. There were no green spots to indicate DNA breaks seen in these cells (Fig. 2E), suggesting that editing was absent in C33A cells and the TALEN pairs selected were very specific to HPV-E7 genes.

### TALEN-mediated editing reduced the expression of E7 in SiHa cells

**Figure 3:**
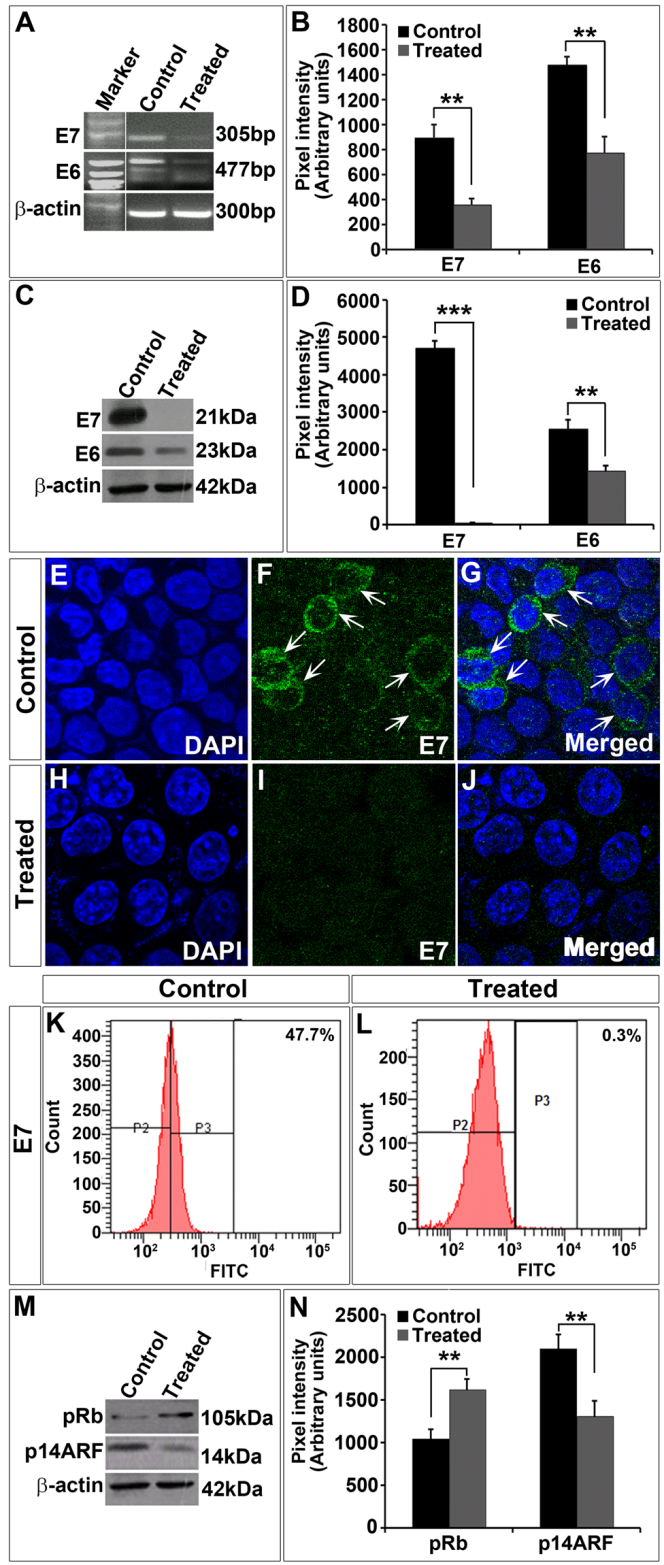
TALEN-mediated E7 editing reduced E7 gene expression in SiHa cells. (**A**) RT-PCR analysis for the transcripts corresponding to *E7* and *E6* showed a significant reduction in treated (TALEN-transfected) group when compared to controls. (**B**) Graph represents expression levels of *E7* and *E6* in control and treated groups. RT-PCR data was further corroborated by western blot analysis which showed a significant reduction in the levels of both E7 and E6 after TALEN treatment (**C**). Graph depicts the densitometric analysis of western blots of E7 and E6 against β-actin protein levels (**D**). Immunocytochemical analysis also indicated that E7 protein levels were significantly decreased in TALEN-treated SiHa cells (**H**–**J**) when compared to controls (**E**–**G**). Further, analysis of E7 expressing cells by flow cytometry also revealed a decrease in the percentage E7 expressing cells in TALEN-treated group (**L**) than in controls (**K**). Western blot analysis of pRb and p14ARF suggested that the levels of pRb were increased and that of p14ARF was reduced in TALEN-treated cells when compared to controls (**M**). The densitometric analysis of the western blots was given in graph (**N**). Data are expressed as mean ± SD from triplicates of three different experiments. **P < 0.001, ***P < 0.0001.

Next, we wanted to check the levels of E7 in response to TALEN effect since gene knockout causes decrease in the levels of transcripts as well as proteins. To prove this, we first carried out an RT-PCR analysis for *E7* in SiHa cells. We observed a significant decrease in the levels of transcript corresponding to *E7* in treated group when compared to controls (Fig. 3A and B). All the controls that we used in the transfection experiments were transfected with vector alone. To check the specificity, we checked the levels of *E6* transcripts as well. Though *E6* also showed a decrease in treated group when compared to controls, it was not as significant as the change we observed in the case of E7 (Fig. 3A and B and Supplementary Fig. 3). When compared to E7, down-regulation of E6 was relatively less. Moreover, E6 and E7 are expressed bicistronically, therefore targeting E7 could have affected E6 expression as well. Since we found a significant reduction in the transcript levels, we sought to check the levels of proteins by Western blot analysis. Our data showed a complete abrogation of E7 proteins in TALEN-treated groups (Fig. 3C and D). The levels of E6 were also found to be decreased (~0.5 fold). Immunocytochemical analysis of E7 proteins also indicated a significant reduction in E7 expression in TALEN-treated cells (Fig. 3H–J) compared to those in control group (Fig. 3E–G). This was further confirmed by FACS analysis with E7-immunostained cells which showed that while 47% of FITC stained E7 was present in control (Fig. 3E and H), only 0.3% of FITC stained E7 was present in TALEN-treated (Fig. 3K) SiHa cells. The FACS data was normalized with secondary antibody controls for E7 (Fig. 3L). Thus, these observations substantiated that TALENs could effectively reduce the transcript as well as protein levels of E7 in SiHa cells.

Our next question was what happens to the interacting partners of E7 in HPV-integrated cells. To address this, we verified the levels of two immediate effector/interacting molecules such as pRb and p14ARF. HPV16-E7 protein is known to destabilize pRb leading to its degradation^19^ whereas E7 is known to up-regulate the expression of p14ARF and p16 in cervical cancer cells^20^. In order to test whether E7 editing by TALENs could affect the downstream targets, we did a western blot analysis for both pRb and p14ARF proteins in TALEN-transfected SiHa cells. As we expected there was a significant increment (~2.5 fold) in the levels of pRb with parallel decrease (~0.5 fold) in the p14ARF levels in the treated cells than controls (Fig. 3M and N). pRb levels can affect the growth of the cells since they act as negative regulators of cell cycle. Together, our data suggest that TALEN-mediated editing of E7 in SiHa cells resulted in a decrease in the E7, both at transcript as well as protein levels which in turn altered the levels of pRb and p14ARF, two interacting partners of E7.

### TALEN-mediated E7 editing in SiHa cells led to cell death by necrosis, and not by apoptosis

**Figure 4:**
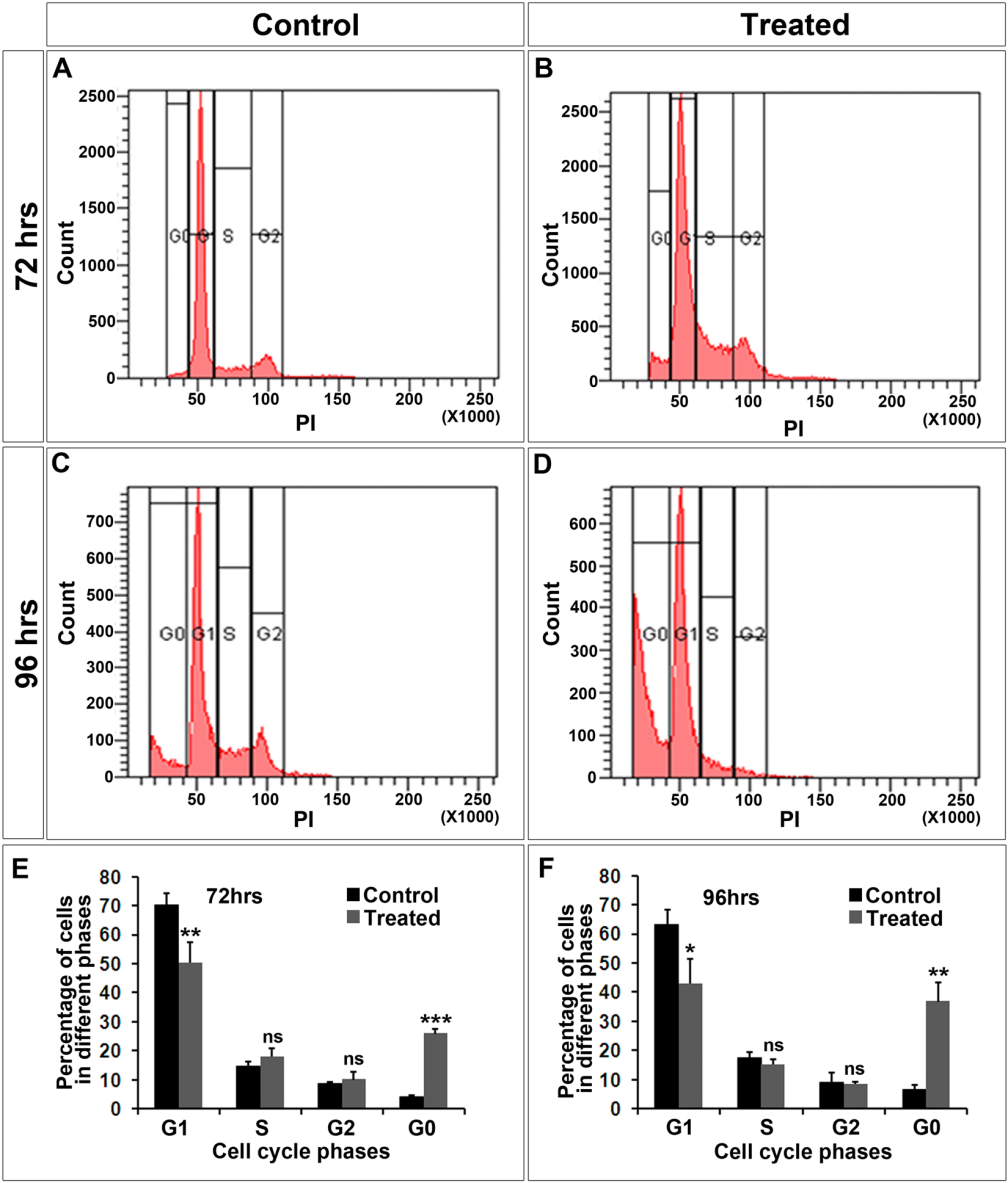
TALEN-treated cells undergo cell death as assessed by Propidium Iodide staining. FACS analysis of PI stained SiHa cells revealed that most of the TALEN-treated cells showed G1/S arrest. (**A**) Represents the FACS profile of control cells at 72 hours, (**B**) TALEN treated SiHa cells at 72 hours, (**C**) SiHa control at 96 hours, (**D**)TALEN treated SiHa cells at 96 hours. (**E**,**F**) Graphs depicting the percentage of cells in each stages of cell cycle during both time intervals of treatments. Data are expressed as mean ± SD from triplicates of three different experiments. ns = not significant, *P < 0.05, **P < 0.001, ***P < 0.0001.

Since we found an increased pRb levels in the TALEN-transfected cells, we assumed that this could lead to cell death. In order to check if TALEN edited cells underwent cell death, cell cycle analysis was performed using Propidium Iodide (PI) staining. PI staining revealed that at seventy two hours, most of the treated cells showed G1/S arrest (Fig. 4B and E) when compared to controls (Fig. 4A and E) and by 96 hours a significant cell death was seen in the treated group (Fig. 4D and F) than in the case of controls (Fig. 4C and F), suggesting that edited cells follow cell death within a time interval of 96 hours.

**Figure 5:**
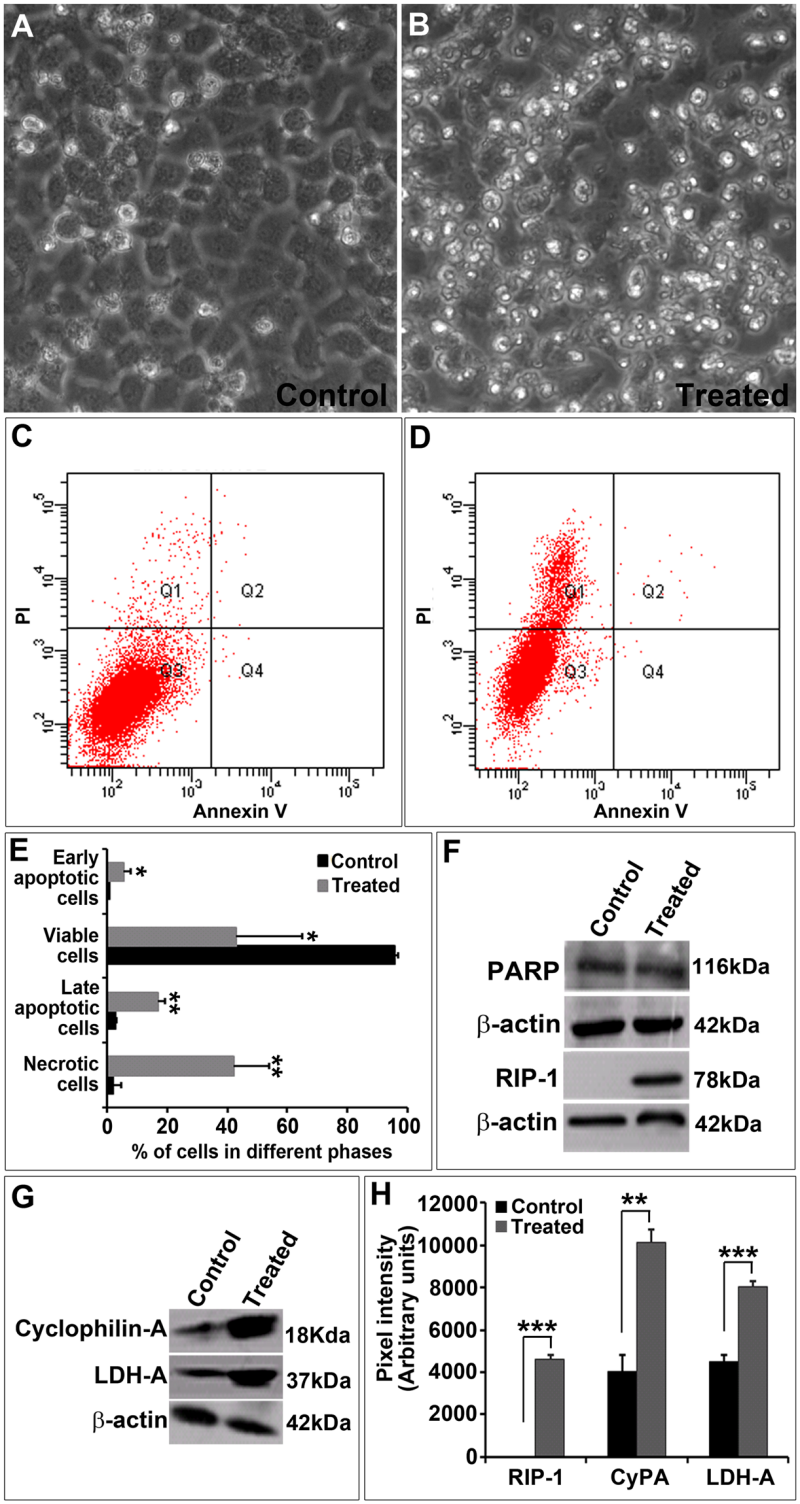
TALEN-mediated E7 editing in SiHa cells led to cell death by necrosis. Bright field microscopic images of (**A**) SiHa control and (**B**) TALEN treated cells. Morphological changes resembling necrosis were observed in SiHa cells (**B**). Annexin V assay in SiHa control showing 5.2% cell death in quadrant 1 (**C**), and TALEN treated cells showed 30.1% cell death in quadrant 1 (**D**) which corresponds to necrotic cell death. (**E**) Graph representing the percentage of cells in different phases. Lysates of TALEN-treated and untreated SiHa cells were immunoblotted with antibodies to PARP revealed absence of cleavage indicating that cells did not undergo apoptosis (**F**). But there was a strong expression of RIP-1 which is a marker of necrosis (**F)**. Immunoblotting with antibodies against Cyclophilin A and LDH-A also pointed to that the TALEN-mediated E7 editing induced necrosis in SiHa cells (**G**). Graphical representation of the proteins levels of RIP-1, CyclophilinA and LDH-A in control and TALEN-treated SiHa Cells (**H**). Data are expressed as mean ± SD from triplicates of three different experiments. *P < 0.05, **P < 0.001, ***P < 0.0001.

The cells treated with TALENs targeting E7 also showed abnormal morphology which resembled necrotic population as observed in the bright-field image taken at 100X. Interestingly, most of the cells in treated group showed a necrotic appearance (Fig. 5B) compared to controls (Fig. 5A). This led us to believe that TALEN-edited cells might be following a necrotic pathway for cell death, rather than apoptosis as reported by a few groups^21–24^. Since we observed significant cell death at 96 hours post treatment by cell cycle analysis, we next sought to do Annexin V staining to determine the exact mechanism of cell death. FACS analysis of Annexin V-stained cells indicated that most of the dead cells corresponded to necrotic population in TALEN-treated group (Fig. 5C and E) as compared to control (Fig. 5D and E). To further rule out apoptosis, we checked the cleavage of PARP. We were unable to detect any significant levels of cleaved PARP (Fig. 5F), which confirmed that edited cells do not follow apoptosis as mode of cell death. Since the treated cells morphologically resembled necrotic cells, we further analyzed the possibility of necrosis as the reason for cell death. For this, we evaluated levels of markers which define necrosis. There was an up-regulation of markers such as RIP-1, Cyclophilin-A and LDH-A (Fig. 5G and H). We observed an approximately 2-fold increase in Cyclophilin-A and 2.5-fold increase in the expression of LDH-A in TALEN-treated group than in controls, thus proving that TALEN-treated cells undergo cell death by necrosis.

### Necrostatin treatment inhibited TALEN mediated cell death by necrosis

**Figure 6:**
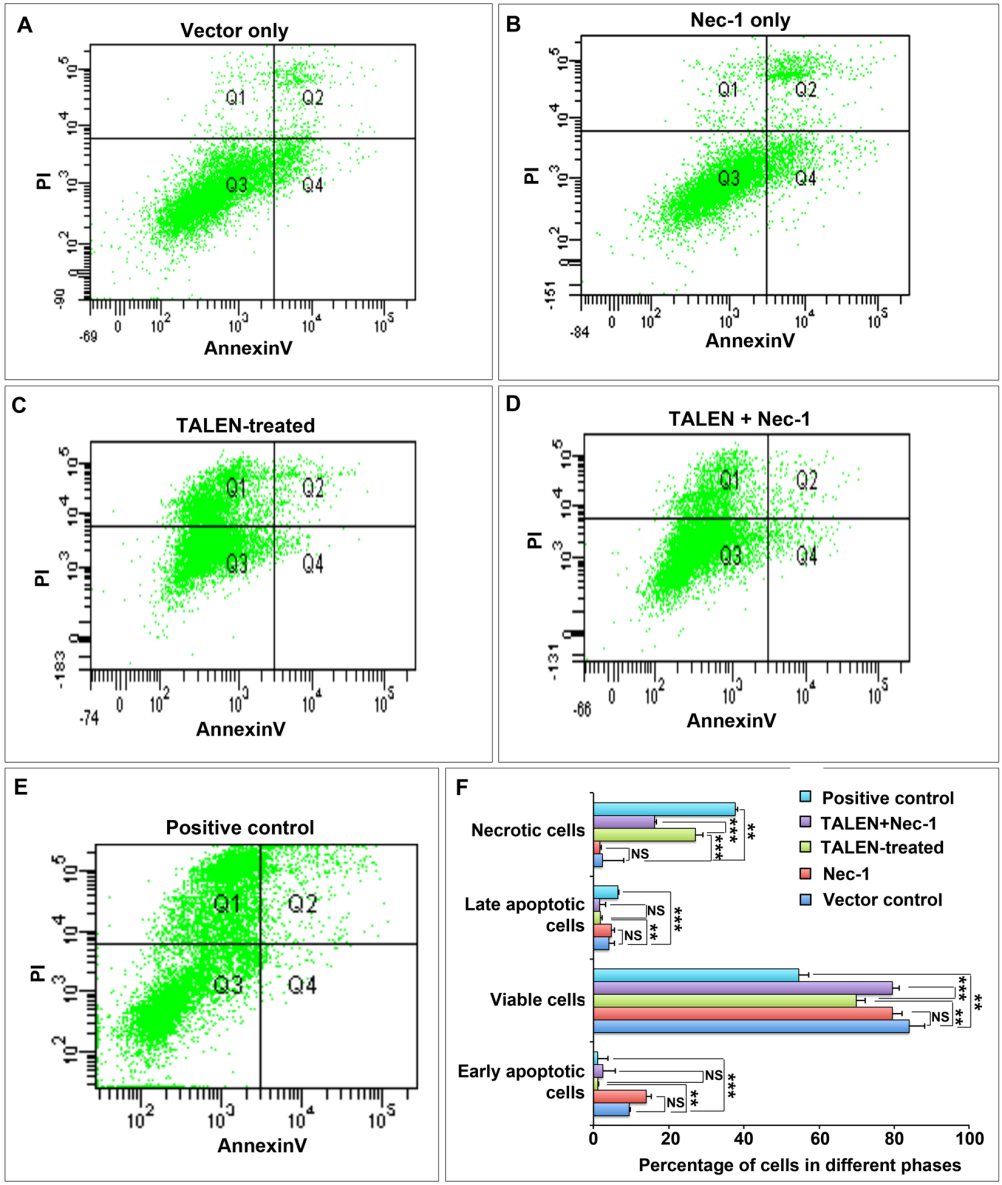
Inhibition of Necrotic cell death in TALEN treated cells by Necrostatin-1. In order to confirm the cell death due to necrosis, we transfected SiHa cells with TALENs and exposed to 50 μM Nec-1 and Annexin V assay was performed using FACS. Annexin V FACS profile of vector alone control (**A**), Nec-1 alone (**B**), TALEN-treated (**C**), TALEN + Nec-1 (**D**) and H_2_O_2_-treated positive controls (**E**). Graph represents the percentage of cells in various phases. Data are expressed as mean ± SD from triplicates of three different experiments. NS-not significant, **P < 0.001, ***P < 0.0001.

In order to further confirm whether the cell death caused by TALEN is due to necrosis, we used necrosis inhibitor, Necrostatin-1 (Nec-1) to negate the effect. Briefly, the cells were exposed to Nec-1 (50 µM) after transfecting with TALENs. Annexin V analysis revealed that the percentage of cells that died due to necrosis was significantly reduced after Nec-1 treatment (Fig. 6D and F) when compared to TALEN-treated group (Fig. 6C and F). As previously observed necrotic cell death was significantly high in TALEN-treated (Fig. 6C and F) and H_2_O_2_-treated positive control (Fig. 6E and F) when compared to vector alone (Fig. 6A and F) and Nec-1 alone (Fig. 6B and F) treated controls. This suggested that indeed the cell death we observed in TALEN-treated cells was mainly due to necrosis.

Our study thus suggests that the selected TALEN pairs could effectively impart editing of HPV-E7 gene in cervical cancer cells thereby inducing the edited cells to undergo cell death mediated by necrosis.

## Discussion

Cervical cancer is one of the most common cancers in women worldwide and in more than 90% of the cases HPV has been found as the principal etiological agent. We therefore found it relevant to use TALEN-based genome editing tool, to disrupt viral genes in the genome, as an alternate therapeutic approach. TALEN targeting E7 showed good activity in our study. Our results showed nearly 10% editing activity and complete silencing of E7. Similarly, TALEN editing efficiency of around 10% was shown in HeLa cell line where a complete abrogation of E7 expression was observed^25^. Tools used to design TALENs aid researchers in targeting specific regions of the gene. While TALEN-T, E-TALEN and Mojo hand design TALENs based on genomic context, restriction enzymes and off- target effects, SAPTA predicts TALEN activity based on RVD composition and site length, restriction enzymes^26^. Hence this tool was appropriate to use to design TALENs. Scores of various monomer sites yielded a composite score that was ranked and TALENs with a score over 40 were chosen that predicted good activity^27^.

The mode of cell death followed by TALEN-mediated editing has been reported to be either by apoptosis or senescence. But we, for the first time, report that necrosis is involved in TALEN-mediated E7 targeting in cervical cancer cells. It was shown earlier that ZFN targeting E7 led to growth inhibition and apoptosis in HPV positive cells^25^. Similarly, E7 and E6 knockout by CRISPR/Cas system also showed inhibition of cellular proliferation and apoptosis and up-regulation of pRB^22–24, 28^. E7-transformed keratinocytes that were knocked out by CRISPRs showed apoptosis^29^. In a study by Hu *et al*.^25^, TALEN targeting E7 also showed apoptosis. Several siRNA designs targeting different regions of the two oncogenes to knockdown E6 or E7 have shown different pathways such as senescence, cell cycle arrest, autophagy and apoptosis^30, 31^. Reports suggest that several factors could affect the difference in death mechanisms that are observed in TALEN-mediated gene editing such as TALEN spacer region, and selection of target region. Necrosis with plasma membrane damage was reported as the mode of cell death in cervical cancer cell lines after photodynamic therapy^32^. Our study also indicated necrotic cell death which could possibly activate pro-inflammatory cytokines leading to tumor-specific immunity. Advantage of necrosis is that it activates and recruits the defense system of the host to the site of damage or invasion^33^. This is in turn activates pro-death signal transduction pathways and doesn’t allow the evolution of treatment resistant cancer cell clones^34^.

In general, viral vectors have been used to deliver ZFNs. However, it has been shown that TALEN delivery is hampered by viral vectors due to presence of repetitive elements in the TALEN framework. Genetic re-arrangements were observed in lentiviral vectors^35^. Another drawback is TALEN size that limits its packaging into these viral vectors^36^. Apart from this, upon infection with viral vectors, both vectors must provide equal quantities of protein. This in turn, would require increased viral dose which could enhance the toxic effects and immune response^37^. The limitations of viral vectors suggest that transient delivery of these molecules could serve as potential adjuvant therapy.

TALENs can be delivered in mRNA transcript form or as proteins which would have several advantages such as (a) avoiding insertional mutagenesis, (b) less off-target effects, (c) low toxicity and (d) fewer regulatory concerns^38^. Researchers have focused on the use of type III secretion systems^39^ and cell penetrating peptides^40^ to transiently deliver TALENs and achieve desired effects. Therefore, in the context of HPV, TALENs could be locally delivered to that region and be given as adjuvant therapy along with surgery. Such a topical application into the cervix could be given at regular intervals and has advantages such as (a) low systemic side effects, (b) high concentration of TALEN proteins could be given locally and(c) avoids metabolism and enzymatic digestion when given as oral drug delivery^25^.

**Figure 7:**
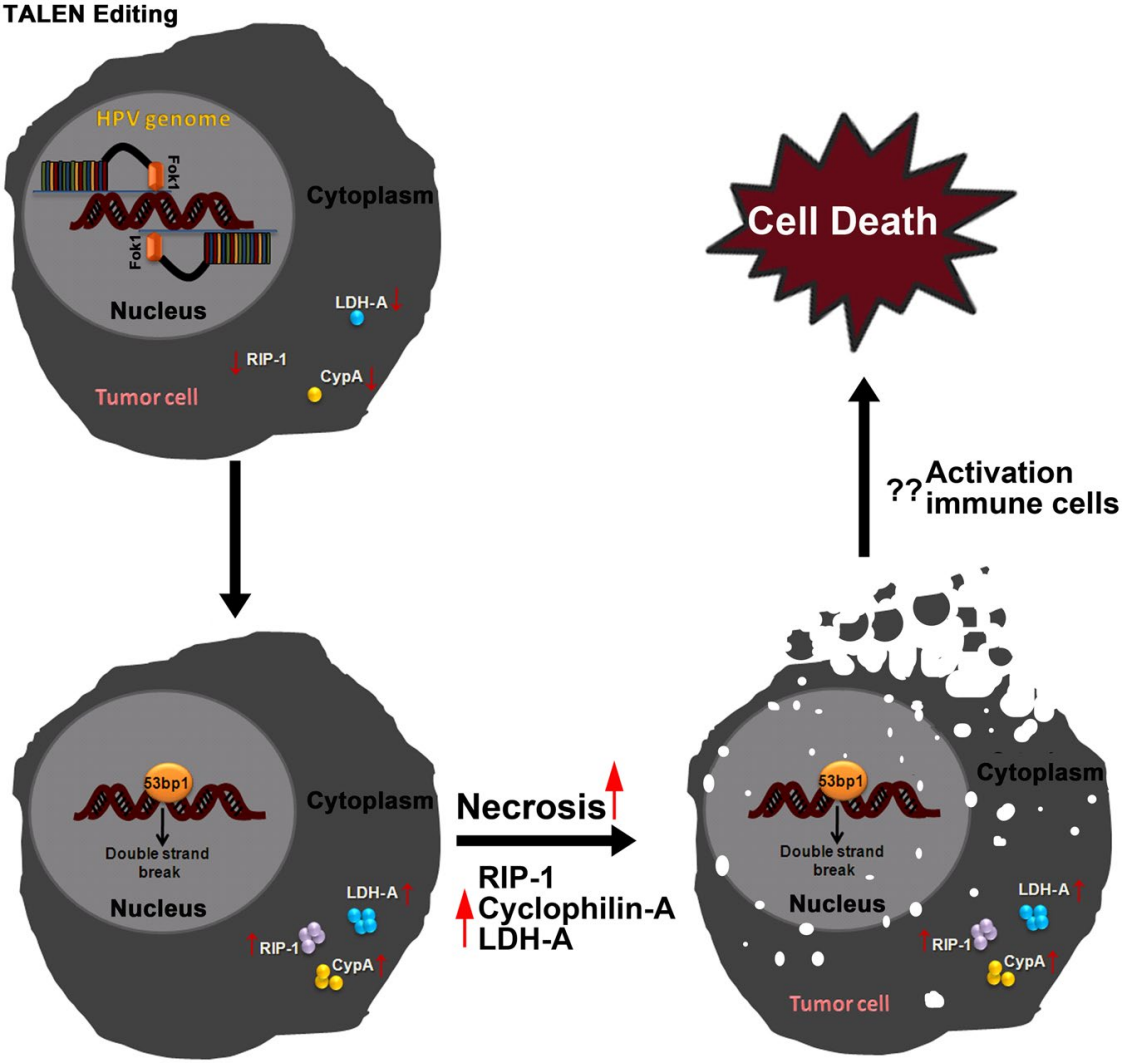
Schematic representation of Necrosis induced by TALEN editing of cervical cancer cells and possible activation of immune cells leading to cell death. When HPV genome integrates in the host genome, it leads to the continuous expression of the two oncogenes E6 and E7. These two oncogenes are responsible for transformation and development of tumor. When TALENs targeting E7 gene is administered (**A**), cells undergo double strand breaks thereby knocking out E7 gene (**B**). Abrogation of E7 gene leads to upregulation of RiP-1, LDH-A, CypA which in turn trigger necrosis (**C**). As an extrapolation of this data, pro-inflammatory cytokines could activate the immune cells and induce cell death. Such a strategy would be useful to induce cell death in cases where tumors acquire resistance to apoptosis through several strategies.

Our study thus suggests that the selected TALEN pairs could effectively impart editing of HPV-E7 gene in cervical cancer cells thereby inducing the edited cells to undergo cell death (Fig. 7). Absence of PARP cleavage and upregulation of necrotic markers such as RIP-1, LDH-A and Cyclophilin-A, confirmed that the cell death induced by TALENs was mediated by necrosis. Such a cell death mechanism would possibly activate the immune system to abolish malignant cells including the cells that are resistant to apoptosis. However, *in vivo* studies need to validate the results obtained in cell lines. Patients generally receive adjuvant therapy in the case of positive lymph nodes, large tumor size, deep stromal invasion and positive surgical margins. Standard chemotherapeutic agents such as cisplatin which are used for adjuvant therapy have been associated with several side effects^41^. Hence, in order to prevent recurrence of cervical cancer after surgery, TALEN based adjuvant therapy could therefore be an option to enhance the effectiveness of primary surgery or radiotherapy for cervical cancer.

## Materials and Methods

### Softwares used for designing TALENs

SAPTA (Scoring algorithm for predicting TALEN activity) was used for predicting TALENs. This online tool (http://bao.rice.edu/Research/BioinformaticTools/TAL_targeter.html) gives an estimate of predicted TALEN activities, allowing selection of optimal target sites among many possible target sites within a given gene segment.

### TALEN assembly and Sequencing

This was done using *Sidansai Biotechnology* TALEN assembly kit (Cat# GL201305-3), according to the vendor’s protocol. Briefly, TALE modules and their backbone vector for right arm and left arm were mixed with solution 1, 2 and 3 and keep the tubes in the thermal cycler with a ligation program 37/25 degrees for 20 cycles. Then the ligation mix was transformed into D18 strain. Colony PCR was done to confirm the positive clones. Plasmids were isolated and sequenced using ABI sequencer (Applied Biosystems 3730 DNA Analyzer).

### Cell culture

SiHa cells were used for our experiment since they are HPV 16 positive cell line and they contain single integrated copy of HPV genome. The cells were cultured and maintained in Dulbecco’s Modified Eagle Medium (DMEM) containing 10% Fetal Bovine Serum (FBS) and 1% Penicillin-Streptomycin. They were maintained at 37 °C in a 5% CO_2_ incubator.

### Transfection in cell lines

Transfection was done using Lipofectamine 3000 reagent (Thermofisher Scientific, Cat No# L3000015) as per manufacturer’s instructions. Briefly, the designed TALEN pair plasmids were isolated using Qiagen midi kit. SiHa cells were seeded at 60% confluency and were transfected with TALEN plasmids (5 µg) using lipofectamine 3000 reagent. Cells were harvested at 72 hours post-transfection and used for further analysis.

### T7E1 Mismatch endonuclease assay

Seventy two hours after treatment with TALENs, genomic DNA was isolated from SiHa cells. PCR for E7 gene was performed in control and TALEN treated samples. Then 10 µl of the PCR products from control and treated samples were taken and subjected to the following program in a thermal cycler (Verito, ABI).

95 °C                                 10 min

95 °C–85 °C            −2 °C/s

85 °C–25 °C            −0.1 °C/s

4 °C                                    ∞

After the reaction was complete, 1 µl of T7E1 enzyme and 2 µl of 10X NEB buffer 2 was added to the PCR samples and incubated at 37 °C for 20 min. The digestion product was run on a 2.0% agarose gel and the result was recorded in the gel documentation system (Bio-Rad). T7E1 assay detects mismatches generated by the NHEJ as cut products against the control which has the PCR product alone. The intensity of the uncut product was analyzed by Image J software to measure TALEN editing activity using the formula X = 100(1−(1 − F)^0.5^ where F is ratio of area under peak of cut and uncut band and X stands for percentage of indels.

### Nuclease resistance assay

This assay is a complementary assay done along with T7E1 assay in order to confirm editing done by TALENs. E7 gene was amplified from DNA extracted from control and treated samples. 5 µl of the PCR product was digested with HPyCh4III enzyme. The interpretation of result is that the control DNA gets completely digested whereas the treated population of cells edited by ZFN show uncut product. The intensity of the uncut product was analyzed by Image J software to measure TALEN editing activity.

### Immunofluorescence

In order to further account for the editing, we assessed the expression of 53BP1, a marker for DNA damage^42^ by immunocytochemical analysis^18^. Thirty six hours after treatment, SiHa cells were washed with PBS and fixed in 4% PFA. Cells were permeabilized with acetone: methanol (1:1) for 20 minutes and blocked with 3% BSA for 1 hour followed by overnight incubation of anti-53BP1 (1:100, Cell signaling Cat# 4937). Cells were then washed with PBS followed by 1-hour incubation with anti-rabbit FITC (1:100, Sigma) and were counterstained with DAPI. Cells were then washed and mounted in 80% glycerol. Images were taken in confocal laser scanning microscope (Nikon).

To confirm E7 expression after TALEN-mediated gene editing post 72 hours after treatment, SiHa cells were washed with 1X PBS and fixed in 4% PFA. Cells were then permeabilized with acetone: methanol (1:1) for 20 minutes and blocked with 3% BSA for 1 hour followed by overnight incubation anti-E7 antibody (1:100, Cat# Sc6981). After washing three times with 1X PBS, the cells were incubated with secondary bodies in appropriate dilutions (1:400, anti-mouse FITC; Life technologies, Cat# A21200). The cells were counterstained with DAPI and mounted in 80% glycerol and sealed. Images were taken in confocal laser scanning microscope (Nikon).

### Western blot analysis

Briefly, SiHa cells were lysed in cell lysis buffer with intermittent vortexing for 1 hour. This was followed by centrifugation of the lysate at 15000 rpm for 10 min at 4 °C and supernatant was collected. The protein samples were quantified using Bradford assay and the volume corresponding to 70 µg of protein was mixed with 4X loading dye and boiled at 95 °C for 7 minutes, then loaded onto a 12% PAGE. Proteins in the gel were transferred onto a PVDF membrane (GE), blocked with 5% milk/PBS-T, and then probed with primary antibodies at appropriate dilutions. Primary antibodies used included: mouse anti-β-actin (1:400, Santacruz, Cat #Sc 47778), mouse anti-E7 (1:400, Santacruz, Cat#Sc- 6981), mouse anti-E6 (1:400, Santacruz, Cat#Sc 460), mouse anti- pRB (1:500, Cell Signalling, Cat# 9309), rabbit anti- p14ARF (1:400 Santacruz, Cat# Sc 53639), rabbit anti-PARP (1:1000, Cell Signalling, Cat# 9542), rabbit anti-RIP1 (1:1000, Cell Signalling, Cat# 4926), rabbit anti-LDHA (1:1000, Cell Signalling, Cat# 2012), Cyclophilin A (1:3000, Abcam, Cat# ab58144). Secondary antibodies included: Goat anti-rabbit HRP (Sigma Aldrich Cat# A9169) and Goat anti-mouse HRP conjugates (Sigma Aldrich, Cat# A4416). Proteins were visualized using chemiluminescence (Immobilon, Millipore). All Western blots were done in triplicates.

### RT-PCR analysis

Isolation of total RNA and cDNA synthesis were carried out as per manufacturer’s instructions. RNA was isolated from the SiHa cells using DNA/RNA kit (Qiagen Cat# 80204) and ~1 μg of RNA was transcribed into cDNA using MMLV reverse transcriptase (Promega Cat# M1701), 0.5 µl of dNTPs, 0.5 µl of RNase inhibitor, 4 µl of reverse random primer in a total volume of 20 μl. Specific transcripts were amplified using gene-specific primers:

*HPV E6*-

Forward: 5′-ATGCATGGAGATACACCTACATTG-3′

Reverse: 5′-CATTACATCCCGTACCCTCTTC-3′;

*HPV E7*-

Forward: 5′-ATGCACCAAAAGAGAACTGCAATGT-3′

Reverse: 5′-TTACAGCTGGGTTTCTCTACGTG-3′;

*β-actin*-

Forward: 5′-AGACTTCGAGCAGGAGATG-3′

Reverse: 5′-CTTGATCTTCATGGTGCTAGG-3′

Amplifications were carried out for 25 cycles on a Veriti Thermal cycler (Applied Biosystems) and the products were visualized by ethidium bromide staining after electrophoresis on 2% agarose gel. The bands corresponding to specific transcripts were scanned using a densitometer and normalized against the values corresponding to *β-actin* transcript bands.

### FACS analysis of E7

FACS was done to quantify the number of cells without E7 expression after treatment with TALEN. For that, SiHa Cells were dissociated using trypsin (0.25%), followed by one wash with IX PBS. The cells were then fixed with 4% PFA for 5 minutes followed by centrifugation at 2500 rpm for 2 minutes. After 3 washes with 1X PBS, cells were incubated with 3% BSA in PBS for 15 minutes and were probed with E7 antibody (anti-mouse 1:100, Santacruz, Cat #SC 6981) in 100 µl at 4 °C for overnight. After sufficient washes with PBS, 100 µl of FITC conjugated chicken anti-mouse antibody (1:100, Santacruz, Cat# A21200) was added to the cells and incubated for 20 minutes at 4 °C. Finally, the cells were washed and re-suspended in 100 µl of 1X PBS, and E7 expression was then analyzed by FACS.10, 000 events were acquired for both control and treated samples. Both control and treated samples had secondary antibody controls.

### Cell cycle analysis

Cells transfected with TALENs were collected at two time points; seventy two hours, ninety six hours, and used for cell cycle analysis. They were washed with PBS, fixed using 70% ethanol (overnight at −20 °C), washed with 1X PBS followed by incubation with 1 µlRNAse A (100 mg/ml Sigma) for 30 min. Cells were then passed through a cell strainer and stained with 10 µl Propidium Iodide (10 mg/mL) by incubating for 10 minutes at room temperature. The stained cells were subjected to flow cytometry (BD FACS Aria). 10,000 events were acquired for both control and treated samples at the above-mentioned time points (Abcam protocol).

### Annexin V assay

In order to check the apoptosis in SiHa cells in response to editing, we performed Annexin V assay. Briefly, SiHa cells were harvested at ninety six hours post-transfection with TALENs and processed for Annexin V staining (FITC Annexin V Apoptosis Detection Kit, Sigma, Cat# APOAF-20T ST) as per vender’s protocol. Necrostatin-1 was used to validate TALEN mediated necrotic death and it was also analyzed by Annexin V assay based on FACS. As a positive control 2 mM H_2_O_2_ was used induce cell death.

Cells were trypsinized (0.25% Trypsin), resuspended in 1 mL PBS, and then centrifuged at 2000 rpm for 5 minutes. Samples were then gently resuspended in 1X binding buffer and centrifuged at 2000 rpm for 5 minutes. They were again gently resuspended in 500 μL of I X binding buffer and passed through a cell strainer. Cells from both control and treated groups were then incubated with 3 μL Annexin V and 10 ul of PI for 10 minutes and were analysed by using a FACS Calibur Aria Flow Cytometer (Becton Dickinson). The blue laser (488 nm) was used for detecting FITC, while the red laser (594 nm) was used for detecting PI. 10,000 events were acquired for both samples.

### Statistical analysis

Results are expressed as mean ± S.E.M of at least three separate experiments. Statistical analyses were done using Student’s t test to determine the significance of the differences between the various conditions.

## Electronic supplementary material

Supplementary information
