# Supplementary material for: TALEN based HPV-E7 editing triggers necrotic cell death in cervical cancer cells
Source: Sci Rep. 2017 Jul 14;7:5500. doi: 10.1038/s41598-017-05696-0 (PMC5511212; doi:10.1038/s41598-017-05696-0)
Supplement: Supplementary file 1 — Supplementary information [file 41598_2017_5696_MOESM1_ESM.pdf]

Supplementary Information

Title: TALEN based HPV-E7 editing triggers necrotic cell death in cervical cancer cells

Sumitra Shankar, Deepti Prasad, Rahul Sanawar, Ani V Das and M. Radhakrishna Pillai*

Cancer Research Program-9, Rajiv Gandhi Centre for Biotechnology, Thycaud.P.O., Thiruvananthapuram-14, Kerala, India

Fig. S1

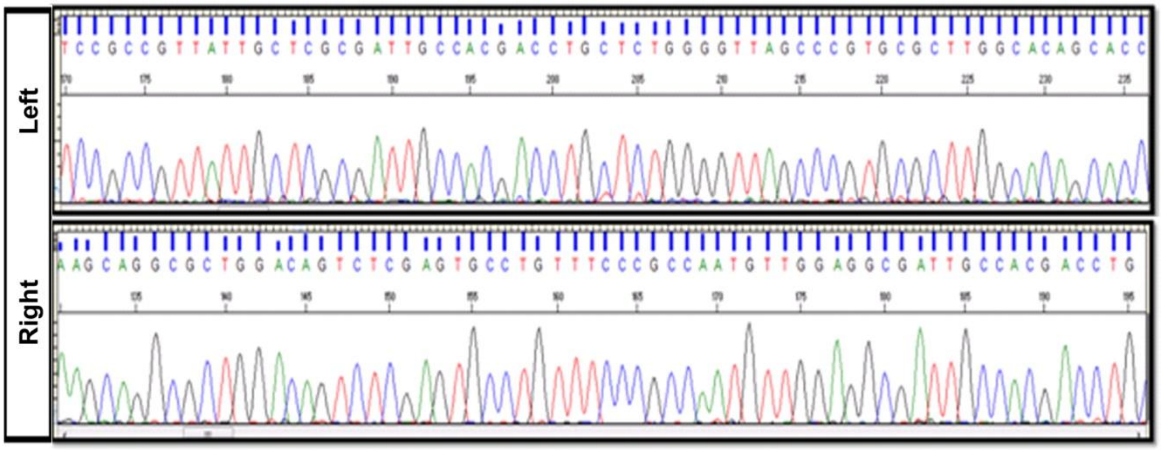

Supplementary Figure legend

Figure S1. **Sequencing data showing right and the left arm of the TALEN pairs.** TALENs were designed using SAPTA program and sequencing analysis showed matching with target sequences at position 44 in the start region of Exon I of E7.

Fig. S2

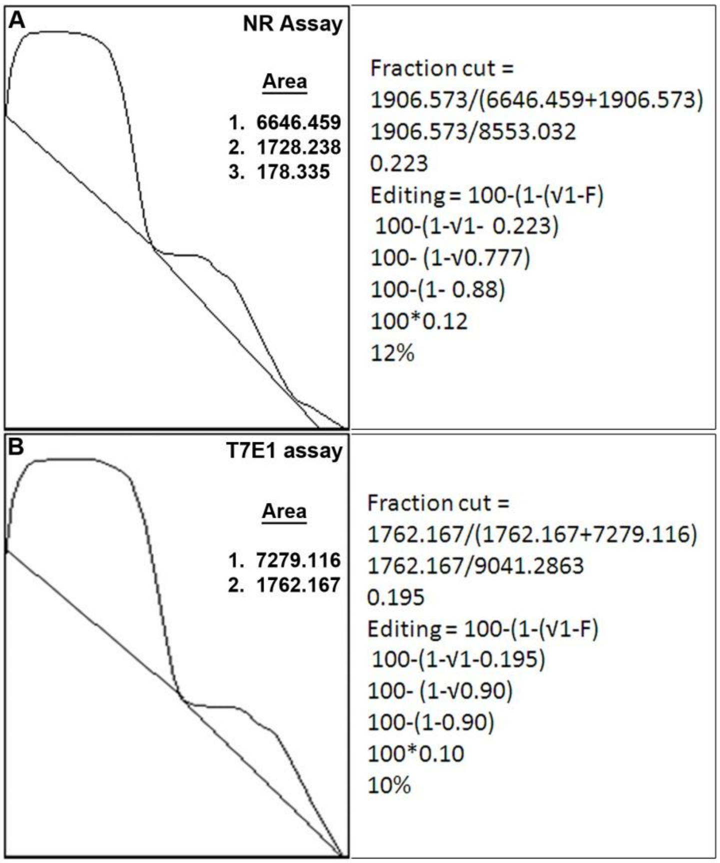

Supplementary Figure legend

Figure S2. TALEN-mediated HPV-E6 editing efficiency in SiHa cells. The gene editing efficiency was calculated using ImageJ software by measuring the intensity of bands which was represented by area under the peak. Area of cut fragment/total area gives a fraction, f, which was then substituted in the formula to get percentage editing [% = 100-(1-(sqrt(1-f))]. As per the calculation, the editing efficiency showed ~12% in Nuclease Resistance (**A**) and 10% in T7E1 (**B**) assays.

Fig. S3

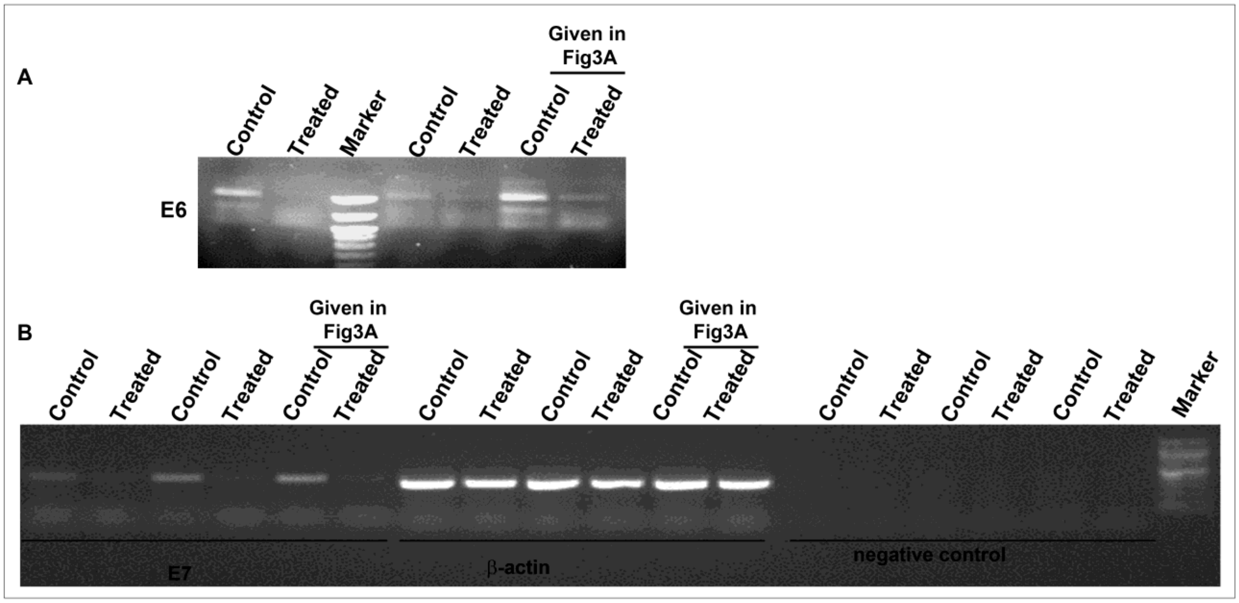

Supplementary Figure legend

Figure S3. Gel images of RT-PCR analysis for E6 and E7 transcripts upon TALEN mediated editing of E7 in SiHa cell lines. Experiments were done in triplicates and the samples were run on the same gel from which the best out the three was given in Figure 3A. (**A**) E6 levels in control and treated groups, (**B**) E7 and β-actin levels in control and treated groups.
